# Reversing wrinkled skin and hair loss in mice by restoring mitochondrial function

**DOI:** 10.1038/s41419-018-0765-9

**Published:** 2018-07-20

**Authors:** Bhupendra Singh, Trenton R. Schoeb, Prachi Bajpai, Andrzej Slominski, Keshav K. Singh

**Affiliations:** 1 Department of Genetics, University of Alabama at Birmingham, Birmingham, AL 35294 USA; 2 Department of Dermatology, University of Alabama at Birmingham, Birmingham, AL 35294 USA; 3 Birmingham Veterans Affairs Medical Center, Birmingham, AL 35294 USA; 4 Center for Free Radical Biology, Center for Aging and UAB Comprehensive Cancer Center, University of Alabama at Birmingham, Birmingham, AL 35294 USA

## Abstract

Mitochondrial DNA (mtDNA) depletion is involved in mtDNA depletion syndromes, mitochondrial diseases, aging and aging-associated chronic diseases, and other human pathologies. To evaluate the consequences of depletion of mtDNA in the whole animal, we created an inducible mtDNA-depleter mouse expressing, in the polymerase domain of POLG1, a dominant-negative mutation to induce depletion of mtDNA in various tissues. These mice showed reduced mtDNA content, reduced mitochondrial gene expression, and instability of supercomplexes involved in oxidative phosphorylation (OXPHOS) resulting in reduced OXPHOS enzymatic activities. We demonstrate that ubiquitous depletion of mtDNA in mice leads to predominant and profound effects on the skin resulting in wrinkles and visual hair loss with an increased number of dysfunctional hair follicles and inflammatory responses. Development of skin wrinkle was associated with the significant epidermal hyperplasia, hyperkeratosis, increased expression of matrix metalloproteinases, and decreased expression of matrix metalloproteinase inhibitor *TIMP1*. We also discovered markedly increased skin inflammation that appears to be a contributing factor in skin pathology. Histopathologic analyses revealed dysfunctional hair follicles. mtDNA-depleter mice also show changes in expression of aging-associated markers including *IGF1R*, *KLOTHO*, *VEGF*, and *MRPS5*. mtDNA-repleter mice showed that, by turning off the mutant *POLG1* transgene expression, mitochondrial function, as well as the skin and hair pathology, is reversed to wild-type level. To our knowledge that restoration of mitochondrial functions can reverse the skin and hair pathology is unprecedented.

## Introduction

Mitochondrial dysfunction is associated with many mitochondrial diseases, most of which are the result of dysfunctional mitochondrial oxidative phosphorylation (OXPHOS). Mitochondrial OXPHOS accounts for the generation of most of the cellular adenosine triphosphate (ATP) in a cell. The OXPHOS function largely depends on the coordinated expression of the proteins encoded by both nuclear and mitochondrial genomes. The human mitochondrial genome encodes for 13 polypeptides of the OXPHOS, and the nuclear genome encodes the remaining more than 85 polypeptides required for the assembly of OXPHOS system. Mitochondrial DNA (mtDNA) depletion impairs OXPHOS that leads to mtDNA depletion syndromes (MDSs)^1, 2^. The MDSs are a heterogeneous group of disorders, characterized by low mtDNA levels in specific tissues. In different target organs, mtDNA depletion leads to specific pathological changes^3^. MDS results from the genetic defects in the nuclear-encoded genes that participate in mtDNA replication, and mitochondrial nucleotide metabolism and nucleotide salvage pathway^1, 4–10^. mtDNA depletion is also implicated in other human diseases such as mitochondrial diseases, cardiovascular^11, 12^, diabetes^13–15^, age-associated neurological disorders^16–18^, and cancer^19–25^.

A general decline in mitochondrial function has been extensively reported during aging^26–33^. Furthermore, mitochondrial dysfunction is known to be a driving force underlying age-related human diseases^16–18, 34–36^. A mouse that carries elevated mtDNA mutation is also shown to present signs of premature aging^37, 38^. In addition to mutations in mtDNA, studies also suggest a decrease in mtDNA content and mitochondrial number with age^27, 29, 32, 33, 39^. Notably, there is an age-related mtDNA depletion in a number of tissues^40–42^. mtDNA depletion is also frequently observed among women with premature ovarian aging^43^. Low mtDNA copy number is linked to frailty and, for a multiethnic population, is a predictor of all-cause mortality^44^. A recent study revealed that humans on an average lose about four copies of mtDNA every ten years. This study also identified an association of decrease in mtDNA copy number with age-related physiological parameters^39^.

To help define the role of mtDNA depletion in aging and various diseases, we created an inducible mouse expressing, in the polymerase domain of POLG1, a dominant-negative (DN) mutation that induces depletion of mtDNA in the whole animal. Interestingly, skin wrinkles and visual hair loss were among the earliest and most predominant phenotypic changes observed in these mice. In the present study, we demonstrate that mtDNA depletion-induced phenotypic changes can be reversed by restoration of mitochondrial function upon repletion of mtDNA.

## Results

### Development of mtDNA-depleter mouse

**Fig. 1:**
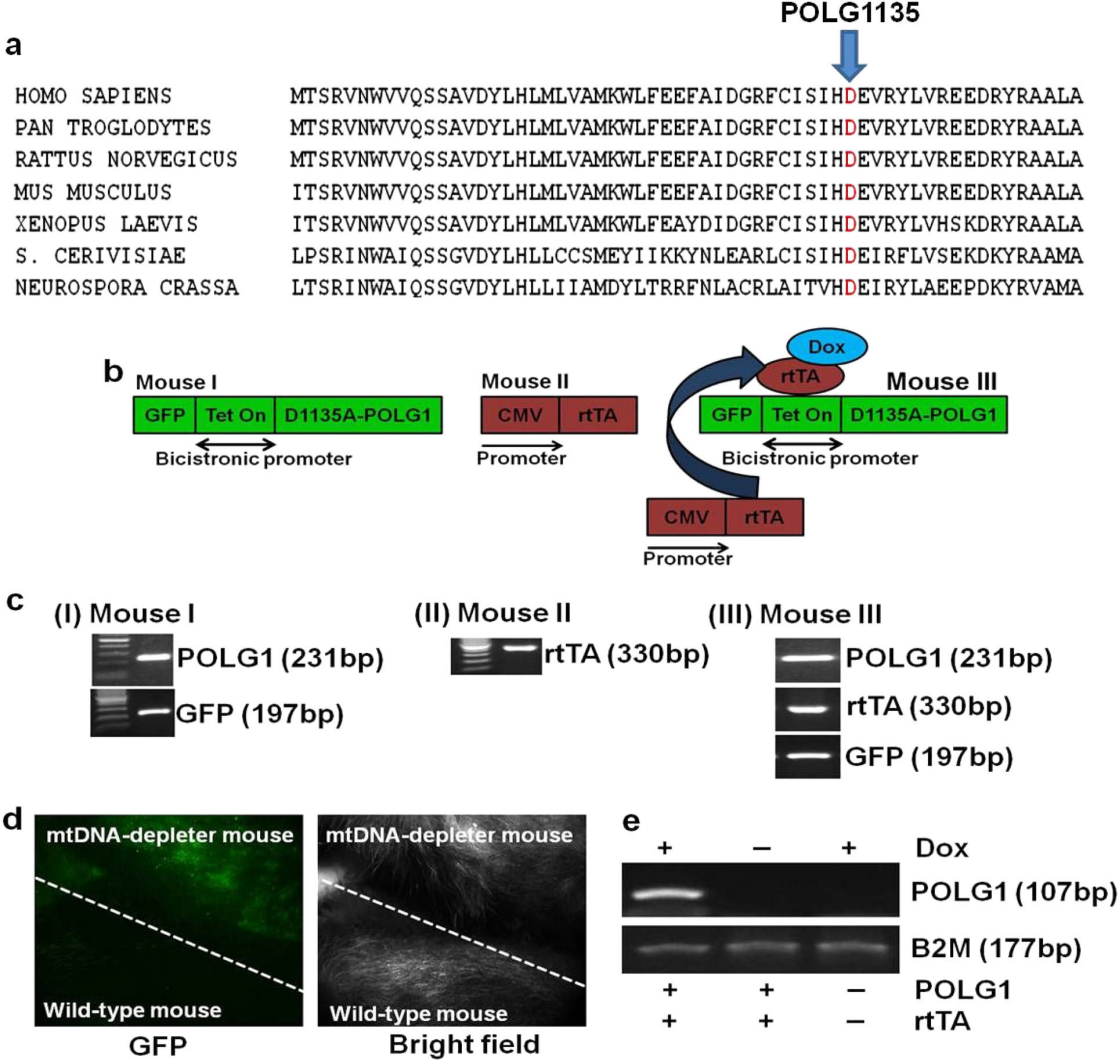
Creation and verification of doxycycline-inducible mtDNA-depleter mice **a** Alignment of amino acid sequences of polymerase domain of POLG1 protein from *Homo sapiens* to *Neurospora crassa* shows that aspartic acid in the POLG1 at 1135 position is evolutionarily conserved. **b** Schematic of the development of inducible D1135A-POLG1 (mtDNA-depleter) transgenic mouse model. D1135A-POLG1-expressing mouse (Mouse I) was created by microinjection of the pTRE-Tight-BI-AcGFP1-D1135-POLG1 construct into the one-cell stage egg from C57BL/6 mouse. The D1135A-POLG1-positive founder male mouse (Mouse I) was bred with the CAG-rtTA3 female mouse (Mouse II, Jackson Laboratories, stock # 016532) to get the D1135A-POLG1 transgenic animal (Mouse III). **c** Pups genotyping reveals the presence of D1135A-POLG1, rtTA, and GFP. **d** Whole-body imaging also confirms expression of GFP in only mtDNA-depleter mice. **e** RT-PCR analyses confirm dox-dependent expression of D1135A-POLG1 in only mtDNA-depleter mice

Aspartic acid to alanine amino acid change at the evolutionarily conserved site in the polymerase domain of POLG1 at 1135 position (D1135A-POLG1) (Fig. 1a) acts as a DN mutation, and its expression leads to decrease in mtDNA content and mitochondrial activity^45, 46^. We developed a Tet-inducible POLG1-DN mouse model with a ubiquitously expressed bidirectional promoter to control the expression of both POLG1-DN and green fluorescence protein (GFP)^46^. POLG1-DN-expressing mouse (Mouse I) was created by microinjection of the pTRE-Tight-BI-AcGFP1-D1135A-POLG1 construct into the one-cell stage egg from C57BL/6 mouse. The POLG1-DN-positive founder male mouse (Mouse I) was bred with the chicken β-actin-reverse tetracycline-controlled transactivator 3 (CAG-rtTA3) female mouse (Mouse II, Jackson Laboratories) to obtain the inducible POLG1-DN transgenic animal (Mouse III) (Fig. 1b). The presence of the DN POLG1, rtTA, and GFP were verified by polymerase chain reaction (PCR) genotyping (Fig. 1c). The rtTA3 was under the control of the ubiquitously expressed cytomegalovirus early enhancer element and CAG promoter. The POLG1-DN transgene was turned on by adding doxycycline (dox) in the food and/or drinking water when the mice were 8 weeks of age. The expression of GFP in POLG1-DN transgenic (mtDNA-depleter) animals was also verified by whole-body imaging for GFP after dox-mediated induction (Fig. 1d). The specificity of dox induction was verified by reverse transcription-PCR (RT-PCR) for the expression of POLG1 in the presence and absence of dox (Fig. 1e).

### Reduced mtDNA, OXPHOS supercomplexes, and enzymatic activities in mtDNA-depleter mice

**Fig. 2:**
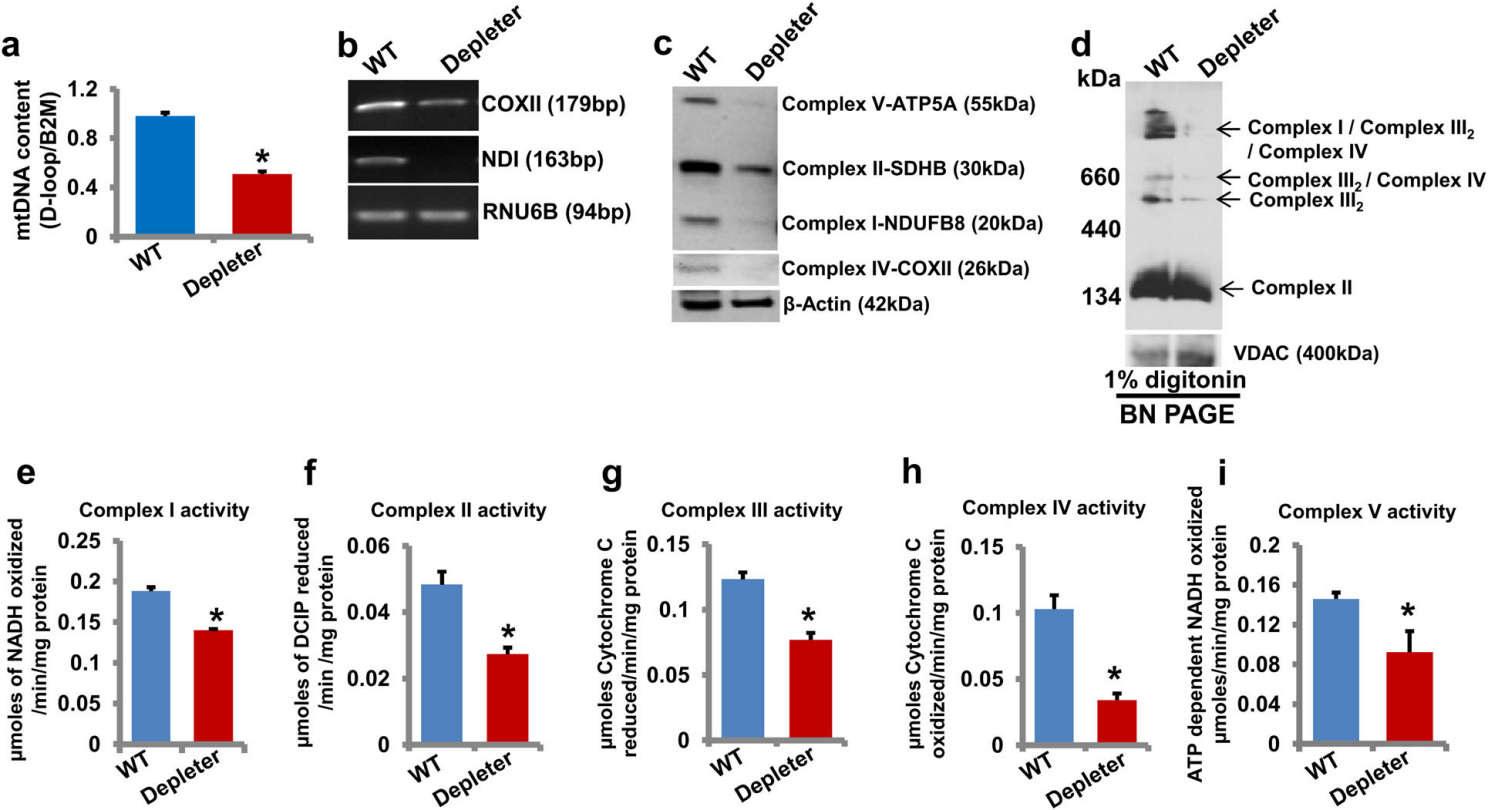
Analyses of mtDNA content, gene expression, and OXPHOS activity in mtDNA-depleter mice **a** Quantification of mtDNA content (mean ± s.e.m; *P<0.05, Student’s *t* test) in skin samples from wild-type control (WT; *n* = 3) and mtDNA-depleter (Depleter; *n* = 3) mice after 2 months of continuous dox induction. **b**–**d** RT-PCR analysis of mtDNA-encoded genes (**b**), Western blot analysis of OXPHOS subunits (**c**), and BN-PAGE analysis of OXPHOS supercomplexes (**d**) in the skin of wild-type control and mtDNA-depleter mice after 2 months of continuous dox induction. **e–i** Enzymatic activities of OXPHOS complex I (**e**), II (**f**), III (**g**), IV (**h**), and V (**i**) in the skin of wild-type control and mtDNA-depleter mice after 2 months of continuous dox induction. Depleter = mtDNA-depleter mice

To further characterize the mtDNA-depleter mice, mtDNA content in different tissues such as the skin (Fig. 2a) and heart, lung, brain, and liver (Supplementary Figure S1) of mtDNA-depleter mice was examined. A significant decrease in mtDNA content in these tissues confirmed the ubiquitous decrease of mtDNA content in mtDNA-depleter mice. mRNA expression of mtDNA-encoded genes (Fig. 2b), expression of OXPHOS proteins (Fig. 2c), and stability of OXPHOS supercomplexes (Fig. 2d) were severely reduced in the skin of mtDNA-depleter mice compared to wild-type littermates. We analyzed enzymatic activities of OXPHOS complexes of mitochondria of the skin of mtDNA-depleter mice. A significant decrease in enzymatic activities of OXPHOS complexes I to V further confirmed mitochondrial dysfunction in mtDNA-depleter mice (Fig. 2e–i). These observations strongly suggest that ubiquitous expression of D1135A-POLG1 leads to reduced mtDNA content, OXPHOS supercomplexes’ stability, and enzymatic activities of OXPHOS complexes in mtDNA-depleter mice.

### mtDNA-depleter mice show inflamed wrinkled skin with the hyperplastic and hyperkeratotic epidermis and alopecia secondary to defective hair loss

**Fig. 3:**
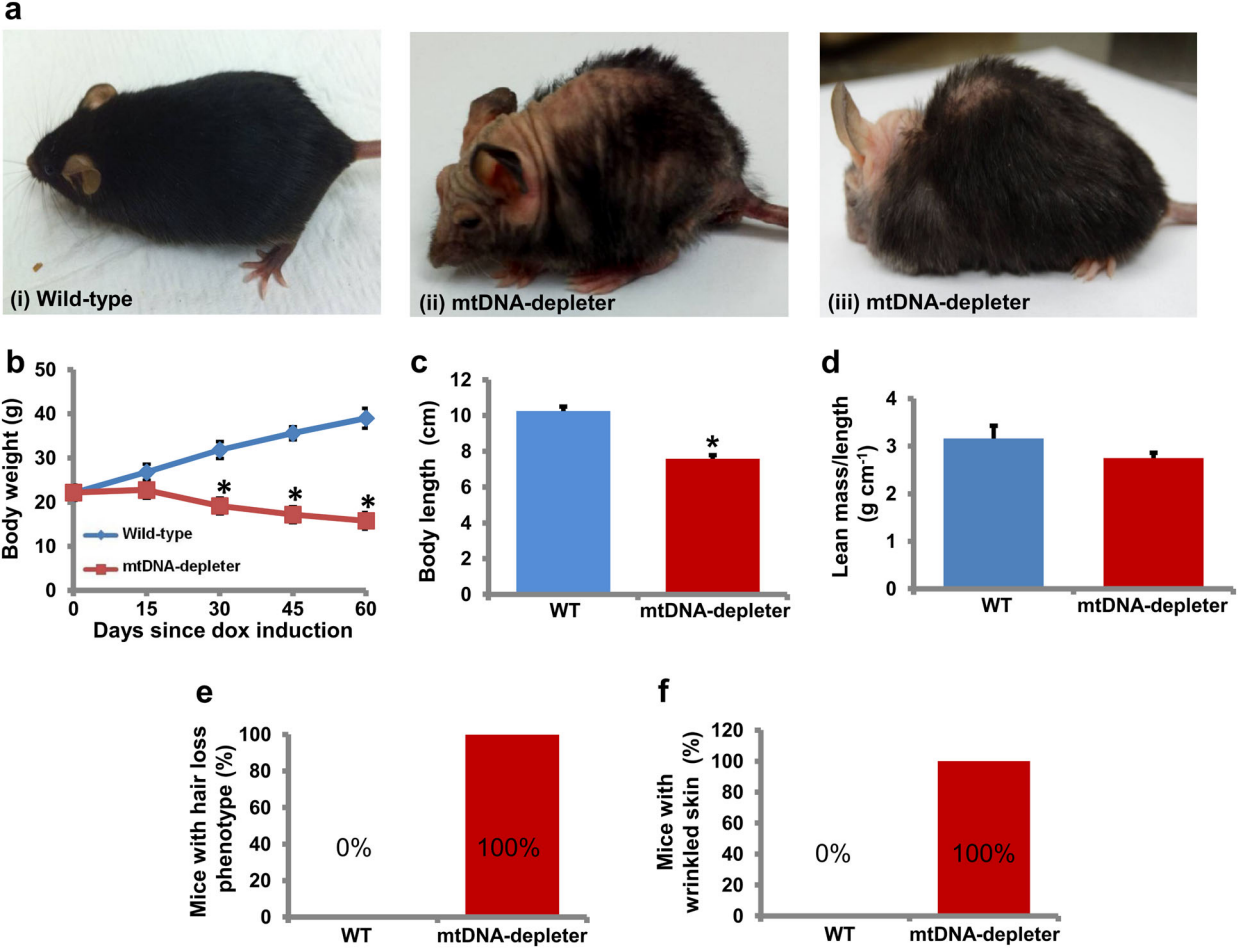
Skin wrinkles and hair loss in mtDNA-depleter mice **a** mtDNA-depleter mice show skin wrinkles (ii), hair loss (ii), and kyphosis (iii) after 4–8 weeks of continuous dox-mediated induction. **b**–**d** Quantitative assessment of body weight (**b**), body length (**c**), and lean mass/length ratio (**d**) of mtDNA-depleter (*n* = 30) and wild-type control mice (*n* = 30). Data are expressed as mean ± s.e.m; *P<0.05, Student’s *t* test. **e**, **f** Quantitative assessment of hair loss (**e**) and wrinkled skin (**f**) phenotypic changes in mtDNA-depleter (*n* = 30) and wild-type control mice (*n* = 30) after 60 days of continuous dox induction

**Fig. 4:**
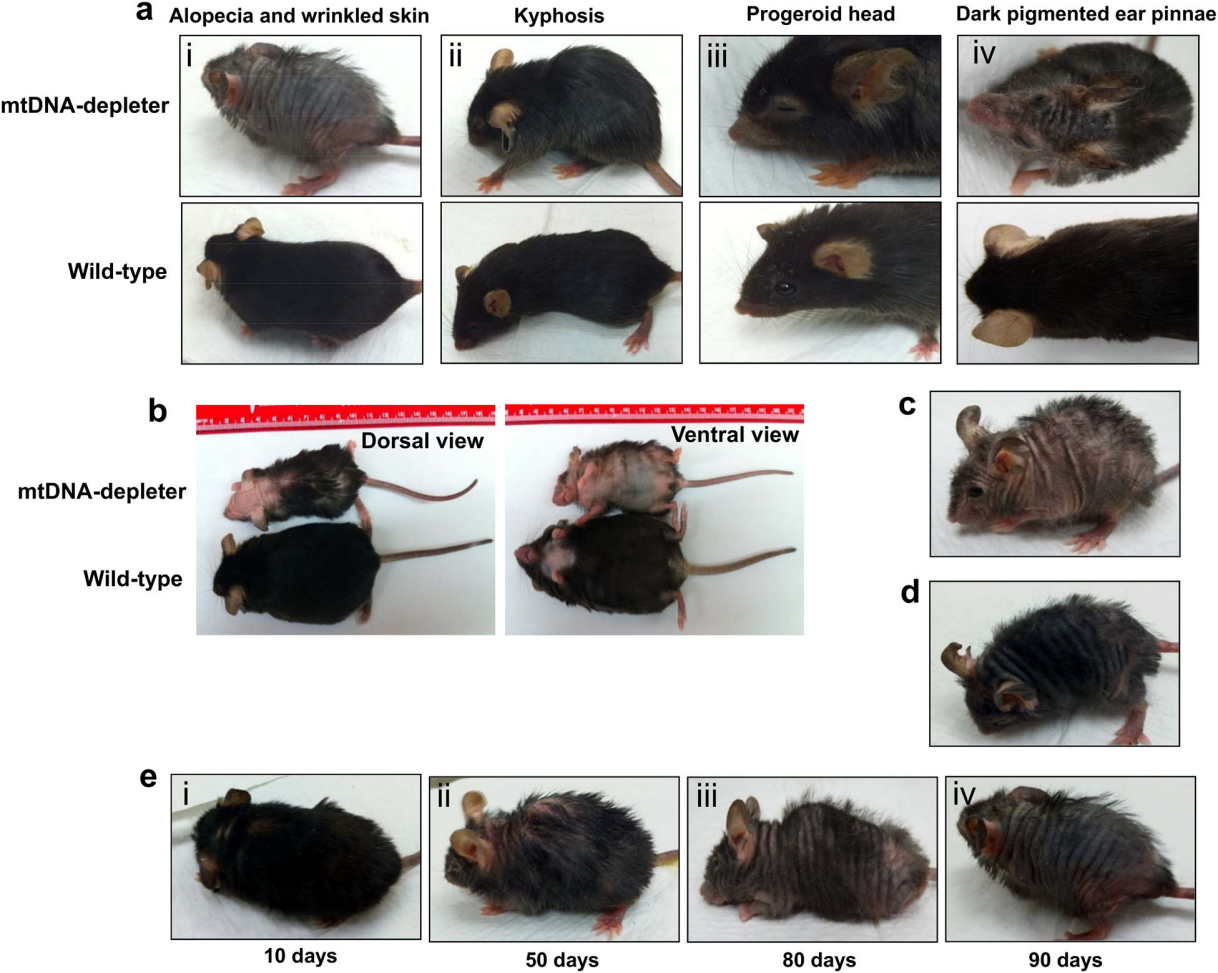
Additional phenotypic changes in mtDNA-depleter mice **a** mtDNA-depleter mice demonstrate a very strong alopecia and wrinkled skin (i), kyphosis (ii), progeroid head (iii), and darkly pigmented ear pinnae (iv) phenotypic changes after induction with dox. **b** Representative images of a mtDNA-depleter mouse showing the gross phenotypic changes in the size and appearance compared to age-matched wild-type control littermate. **c**, **d** The different patterns of hair loss in male (**c**) and female (**d**) mtDNA-depleter mice. **e** Representative images showing gradual time-dependent phenotypic changes in skin wrinkles and hair loss in a female mtDNA-depleter mouse after continuous dox induction (i–iv)

The mtDNA-depleter mice showed a normal appearance until the dox was administered at the age of 8 weeks. After 2 weeks of dox induction, change in scurf was the first phenotypic symptom. After two more weeks with dox induction gray hair, reduced hair density, hair loss (alopecia), kyphosis, progeroid head (Figs. 3a and 4a), slowed movements, and lethargy was the next line of phenotypic changes that are essentially the reminiscent of phenotypic changes naturally occurring during aging^37, 38^. The decrease in size and weight of mtDNA-depleter mice was noticeable at this stage (Figs. 3b, c and 4b). No significant change in lean mass to length ratio was observed between wild-type and mtDNA-depleter mice (Fig. 3d). Continuous induction of POLG1-DN transgene led to the death of some of these mice due to severe mitochondrial malfunction. Fifty percent of the total mtDNA-depleter mice examined in this experiment (*n* = 30) died around 40 days of dox induction, while the remaining mtDNA-depleter mice died within 150 days since initiation of dox induction.

All the mtDNA-depleter mice that survived at least 30 days after dox induction showed the development of alopecia (Fig. 3e). Further extending the duration of dox induction leads to a gradual change in the pattern of hair loss in mtDNA-depleter mice (Fig. 4e). Interestingly, the pattern of hair loss was different in male and female mtDNA-depleter mice. While male mice showed dispersed hair loss (Fig. 4c), females represented time-dependent hair loss patterns and overall more severe hair loss compared to male mice (Fig. 4d, e). Sex hormones regulate mitochondrial functions and may be an underlying mechanism for gender-specific differences observed in hair loss pattern in mtDNA-depleter mice^47^.

**Fig. 5:**
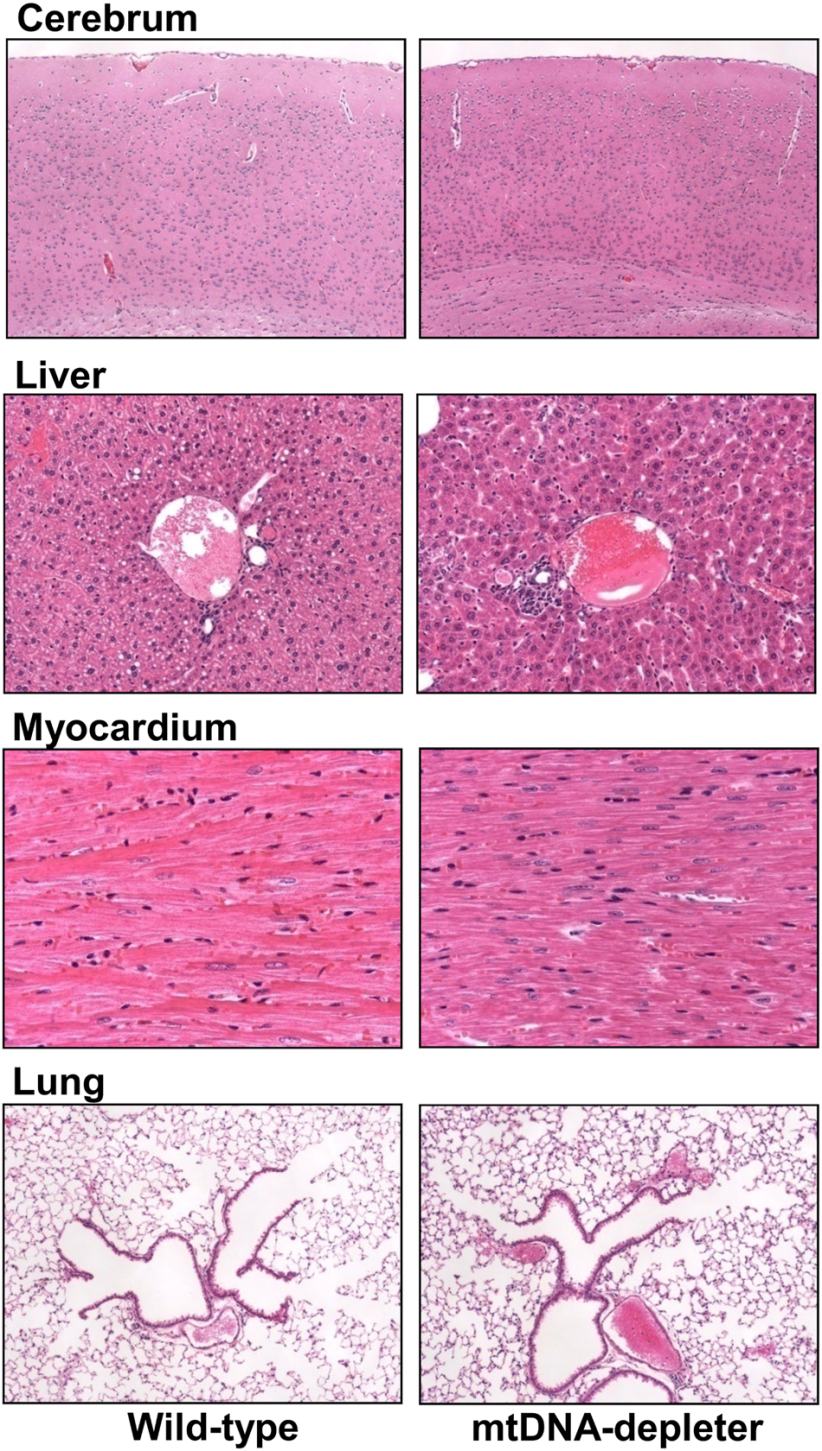
Histological analyses of different tissues from mtDNA-depleter mice Representative hematoxylin- and eosin-stained cross-sections of brain (cerebrum), liver, heart (myocardium), and lung from wild-type control (*n* = 3) and mtDNA-depleter mice (*n* = 3) after 2 months of dox induction

Besides hair loss, skin wrinkles were also evident in all mtDNA-depleter mice (Fig. 3a–f). Female mice exhibited more severe skin wrinkles (Fig. 4d) compared to age-matched male mtDNA-depleter mice (Fig. 4c). We did not notice any phenotypic changes in the wild-type control group fed on dox diet (Fig. 3a), nor in mtDNA-depleter mice without dox diet (normal diet). We conducted a histopathological evaluation of different tissues of mtDNA-depleter mice. Interestingly, no significant histological changes except the reduction in cell sizes were observed in the brain, liver, myocardium, and lung sections of mtDNA-depleter mice after 2 months of dox induction (Fig. 5). Optimal mitochondrial functions are required to maintain the cell size^48^. Thus, the reduced cell size might be an indication of mitochondrial dysfunction in these organs. At both phenotypic and histological levels, the skin was the first and most affected organ.

**Fig. 6:**
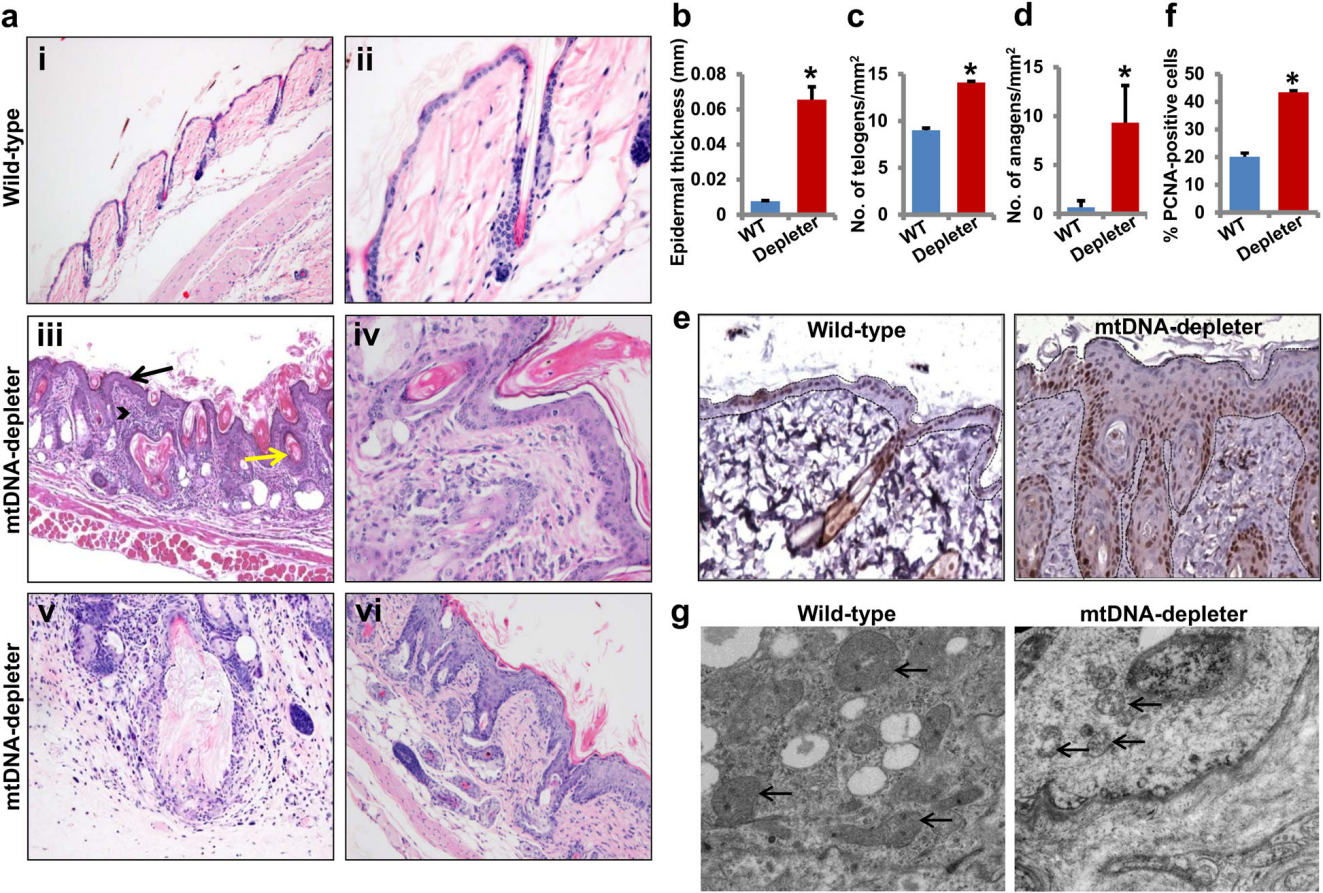
Histological and microscopic analyses of skin of mtDNA-depleter mice **a** Hematoxylin- and eosin-stained sections of dorsal skin from wild-type control (*n* = 3) (i and ii) and mtDNA-depleter mice (*n* = 3) (iii–-vi) after 2 months of continuous dox induction. While the skin of wild-type mice shows the presence of normal skin histology (i, ×10), the skin of mtDNA-depleter mice shows hyperplastic epidermis with hyperkeratosis (black color arrow), dysfunctional hair follicles containing keratinaceous debris and/or malformed hair (yellow color arrow), and increased the number of inflammatory cells in the dermis (arrowhead) (iii, ×10). Skin sections at higher magnification show the presence of normal telogen hair follicles (ii, ×40) in wild-type control mice and aberrant telogen (iv, ×40) and anagen hair follicles (vi, ×20) with defective sebaceous glands. Panel v shows ruptured follicular cyst surrounded by granulomatous and mixed inflammatory infiltrate in mtDNA-depleter mice. **b**–**d** Quantification of epidermal thickness (**b**), hair follicles in telogen (**c**), and anagen (**d**) stages of hair cycle (mean ± s.e.m; *P<0.05, Student’s *t* test) in skin samples from wild-type control (*n* = 3) and mtDNA-depleter (*n* = 3) mice after 2 months of continuous dox induction. **e** Representative images of PCNA immuno-stained cross-sections of skin from wild-type control (*n* = 3) and mtDNA-depleter mice (*n* = 3) after 2 months of dox induction. The basement membrane position in these images is marked with dotted lines. **f** Quantification of epidermal proliferation (PCNA^+^) in skin samples from wild-type control (*n* = 3) and mtDNA-depleter (*n* = 3) mice after 2 months of continuous dox induction. **g** Electron micrographs of skin samples from wild-type control (*n* = 3) and mtDNA-depleter mice (*n* = 3) after 2 months of dox induction. Skin from mtDNA-depleter mice revealed a severely disturbed mitochondrial structure with loss of cristae and degeneration of intramitochondrial structures. Depleter = mtDNA-depleter mice

The examination of hematoxylin- and eosin-stained sections of the skin from the wild-type and mtDNA-depleter mice showed striking histological differences in all skin compartments (Fig. 6). The skin from wild-type animals showed typical morphology of telogen skin in which epidermis was thin, composed of 1–2 layers of keratinocytes, dermis was free of inflammatory infiltrate, and the vast majority of hair follicles were at telogen stage (Fig. 6a, panels i and ii)^49, 50^. In striking contrast, the skin from mtDNA-depleter mice had hyperplastic and hyperkeratotic epidermis, with 4–6 layers of keratinocytes being reminiscent of pathological human epidermis composed of stratum basale, spinosum, and granulosum covered by parakeratotic (predominantly) and compact orthokeratotic scale (Fig. 6a, panels iii–vi). This epidermal hyperplasia is further confirmed by increased expression of proliferation marker PCNA (proliferating cell nuclear antigen) in the skin of mtDNA-depleter mice (Fig. 6e, f). Epidermal hyperplasia is one of the common characteristics of extrinsic aging and is associated with wrinkle formation^51–53^. The increased thickness of the epidermis was primarily due to acanthosis and increased the thickness of the stratum spinosum and stratum granulosum, normally not present in mice (Fig. 6b). A considerable hyperkeratosis, including both parakeratosis and orthokeratosis was evident (Fig. 6a, panels iii–vi). The keratinocytic hyperplasia with hyperkeratosis extended into the infundibula of the hair follicles, of which infundibula were occluded by keratotic plugs. This was also associated with formation of follicular cysts, infundibular (epidermoid) type, with some of them ruptured with secondary granulomatous and suppurative inflammation (Fig. 6a, panels iii and v). The majority of the hair follicles showed pathological alterations (Fig. 6a–d). Although there was evidence of follicular cycling and increased number of follicles in both telogen (Fig. 6c) and anagen (Fig. 6d) in mtDNA-depleter compared with wild-type mice, these follicles were aberrant and did not produce normal hair shafts in mtDNA-depleter mice. Instead, follicles contained predominantly keratinaceous debris with only a few developing hair shafts which were fragmented and malformed. Thus, alopecia was not due to loss of hair follicles or cessation of cycling; rather, the follicles were dysfunctional and could not produce normal hair shaft or completely lost this capability. Furthermore, abnormal formation of hypertrophic sebaceous glands was noted (Fig. 6a, panels iii and vi) with some areas reminiscent of nevus sebaceous in the human skin.

To establish a link between the changes in the skin and the mtDNA stress, we analyzed skin samples by electron microscopy. Electron microscopic analyses revealed the presence of severely degenerated mitochondria with loss of cristae in the skin of mtDNA-depleter mice (Fig. 6g). Together, these studies indicate that mtDNA depletion in the whole animal predominantly induces skin wrinkles due to epidermal hyperplasia and hyperkeratosis, and alopecia because of abnormal hair follicle development and the loss of ability to produce hair shafts.

### Skin inflammation in mtDNA-depleter mice

**Fig. 7:**
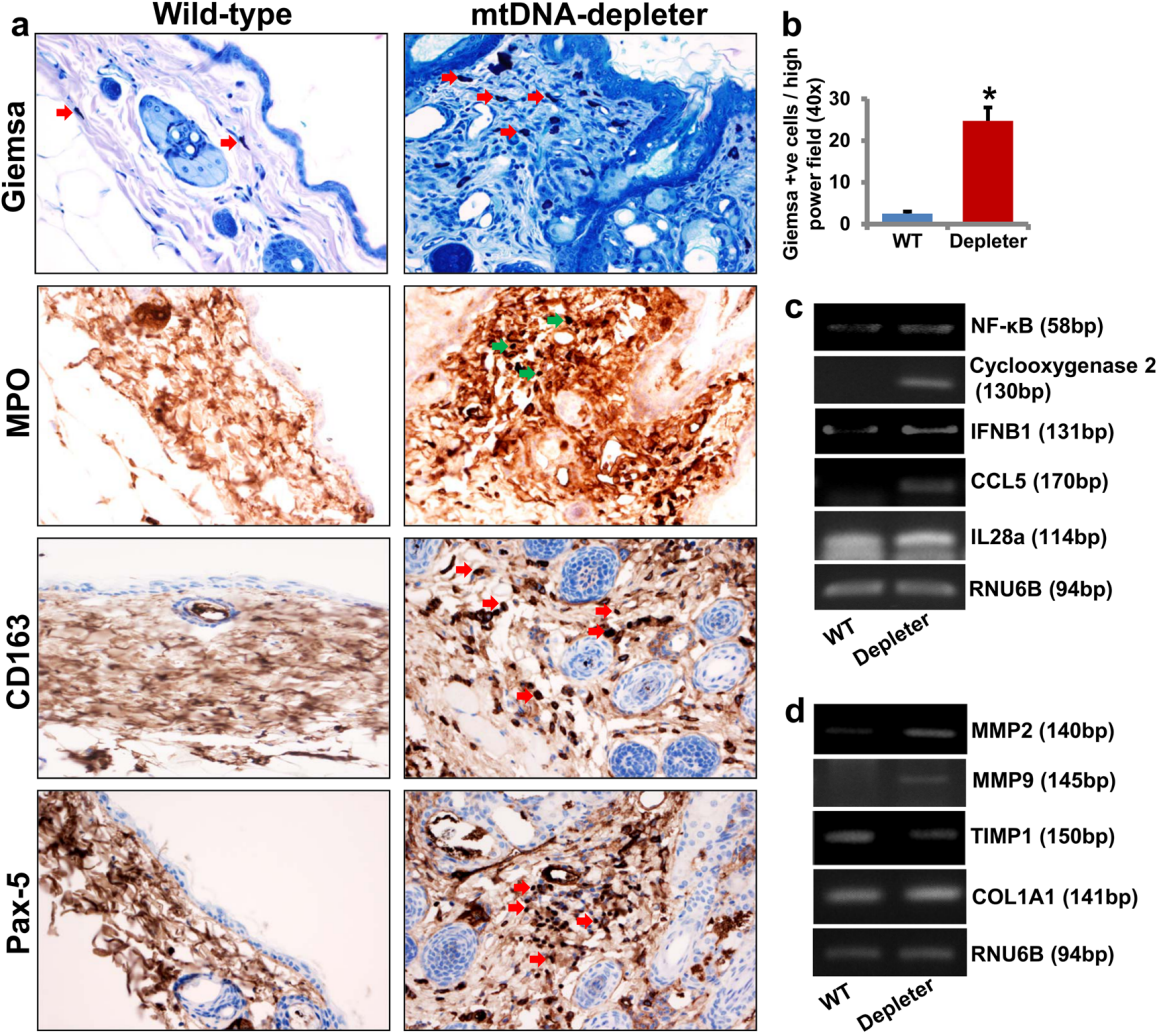
Skin inflammation in mtDNA-depleter mice **a** Immunocytochemical and histochemical analyses of skin sections show the presence of increased number of inflammatory cells including mast cells (Giemsa stain-positive cells), granulocytes (MPO-positive cells), macrophages and histiocytes (CD163-positive cells), and B lymphocytes (Pax-5-positive cells) in the dermis, as well as in perifollicular and periepidermal location of mtDNA-depleter mice. The skin sections of wild-type mice are predominantly negative for MPO, CD163, and Pax-5 staining. Arrows indicate the presence of inflammatory cells in the skin sections. **b** Quantitative analysis of Giemsa-positive mast cells in the skin sections of wild-type control and mtDNA-depleter mice (mean ± s.e.m; *P<0.05, Student’s *t* test). **c** RT-PCR analysis of inflammatory genes in the skin RNA samples of wild-type control (WT; *n* = 3) and mtDNA-depleter mice (Depleter; *n* = 3) after 2 months of continuous dox induction. **d** RT-PCR analysis of genes in the skin RNA samples of wild-type control (*n* = 3) and mtDNA-depleter mice (*n* = 3) after 2 months of continuous dox induction. Depleter = mtDNA-depleter mice

Skin wrinkles are a hallmark of both intrinsic and extrinsic aging of the skin. Alterations in the mitochondrial genome have been associated with the extrinsic aging of the skin^54^. The presence of coarse skin wrinkles with marked acanthosis and inflammatory cells in the dermis of mtDNA-depleter mice presented characteristics akin to the extrinsic aging of skin in human^55^. We examined the skin sections for the presence of inflammatory infiltrate in the skin of mtDNA-depleter mice (Fig. 6a). While control mice showed lack of skin inflammation, the mtDNA-depleter mice showed marked mixed dermal inflammatory infiltrate which were also present to a different degree in epidermal and adnexal structures. The infiltrate was predominantly lymphohistiocytic and contained neutrophils, mast cells, and to some degree eosinophils (Fig. 6a). In the areas where follicular cysts were ruptured, neutrophilic infiltrate accompanied by the granulomatous reaction was predominant. To better define the nature of inflammatory cells, immunocytochemistry and histochemistry were performed. These confirmed presence of increased number of inflammatory cells including mast cells (Giemsa stain-positive cells, Fig. 7a, b), granulocytes (MPO-positive cells, Fig. 7a), macrophages and histiocytes (CD163-positive cells, Fig. 7a), B lymphocytes (Pax-5-positive cells, Fig. 7a), and T lymphocytes (CD3-positive cells, data not shown) in the dermis, as well as in perifollicular and periepidermal location of mtDNA-depleter mice. The skin sections of wild-type mice were predominantly negative for MPO, CD3, CD163, and Pax-5 staining and showed only occasional mast cells. Florid skin inflammatory responses further support the causative link between mitochondrial dysfunction and inflammation^56, 57^. We observed increased expression of inflammatory genes such as *IFNB1*, *IL28a*, and *CCL5* in the skin samples of mtDNA-depleter mice compared to the skin samples of wild-type mice (Fig. 7c). Our study revealed increased expression of *NF-κB* and *Cyclooxygenase 2*, a nuclear factor-κB (NF-κB)-regulated mediator of inflammation in the skin of mtDNA-depleter mice compared to the skin from wild-type littermates (Fig. 7c). These observations suggest that inflammation contributes to the skin aging in mtDNA-depleter mice.

### Altered expression of matrix metalloproteinases in the skin of mtDNA-depleter mice

Skin wrinkling is associated with a loss of collagen fibers^58^. A tight balance between the proteolytical enzymes matrix metalloproteinases (MMPs) and their tissue-specific inhibitor tissue inhibitor metalloproteinase-1 (*TIMP1*) is essential to maintain the collagen fiber content in the skin^59^. Our study revealed increased expression of *MMP2* and *MMP9* and decreased expression of *TIMP1* in mtDNA-depleter mice (Fig. 7d). Expression of collagen type 1 alpha-1 (*COL1A1*), an important gene in the de novo synthesis of collagen of the skin, remained unaltered (Fig. 7d). These studies suggest that skin wrinkling-associated markers are dysregulated in mtDNA-depleter mice.

### Altered expression of markers of aging in mtDNA-depleter mice

**Fig. 8:**
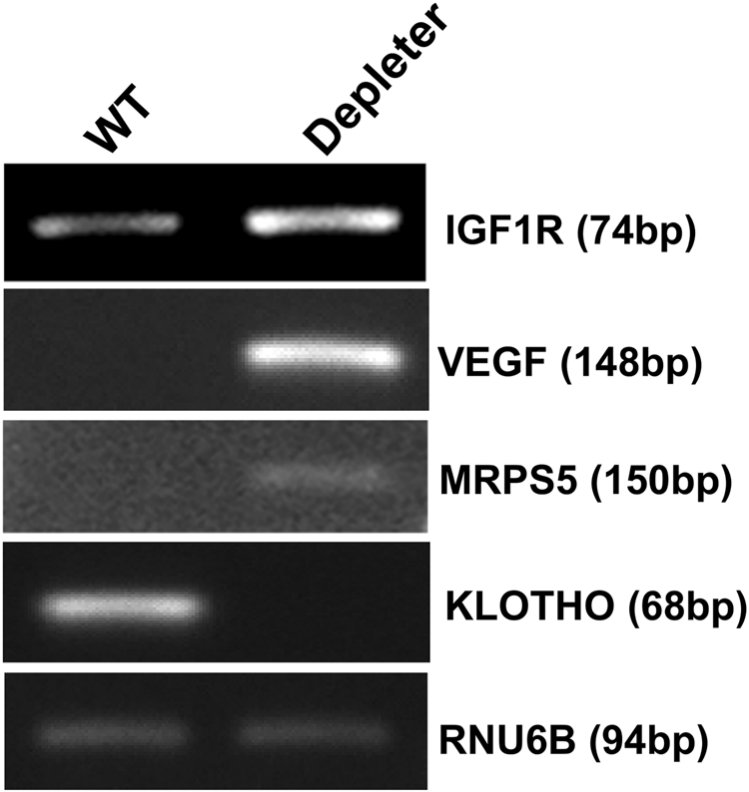
Expression of aging-associated markers in mtDNA-depleter mice Representative images showing mRNA expression analyses of *IGF1R*, *VEGF*, *MRPS5*, and *Klotho* genes (marker genes of intrinsic aging) by RT-PCR in the skin samples of wild-type control (*n* = 3) and mtDNA-depleter mice (*n* = 3) after 2 months of dox induction. Depleter = mtDNA-depleter mice

To characterize the association of skin wrinkles and aging at the molecular level, we analyzed expression of markers related to intrinsic aging in the skin of mtDNA-depleter mice. Increased expression of molecular markers of intrinsic aging like *IGF1R*, *VEGF, MRPS5* and decreased expression of *Klotho* suggested towards intrinsic aging in mtDNA-depleter mice (Fig. 8)^60–62^. These observations suggest that mitochondrial dysfunction induces skin aging.

### Reversal of wrinkled skin and loss of hair by repletion of mtDNA

**Fig. 9:**
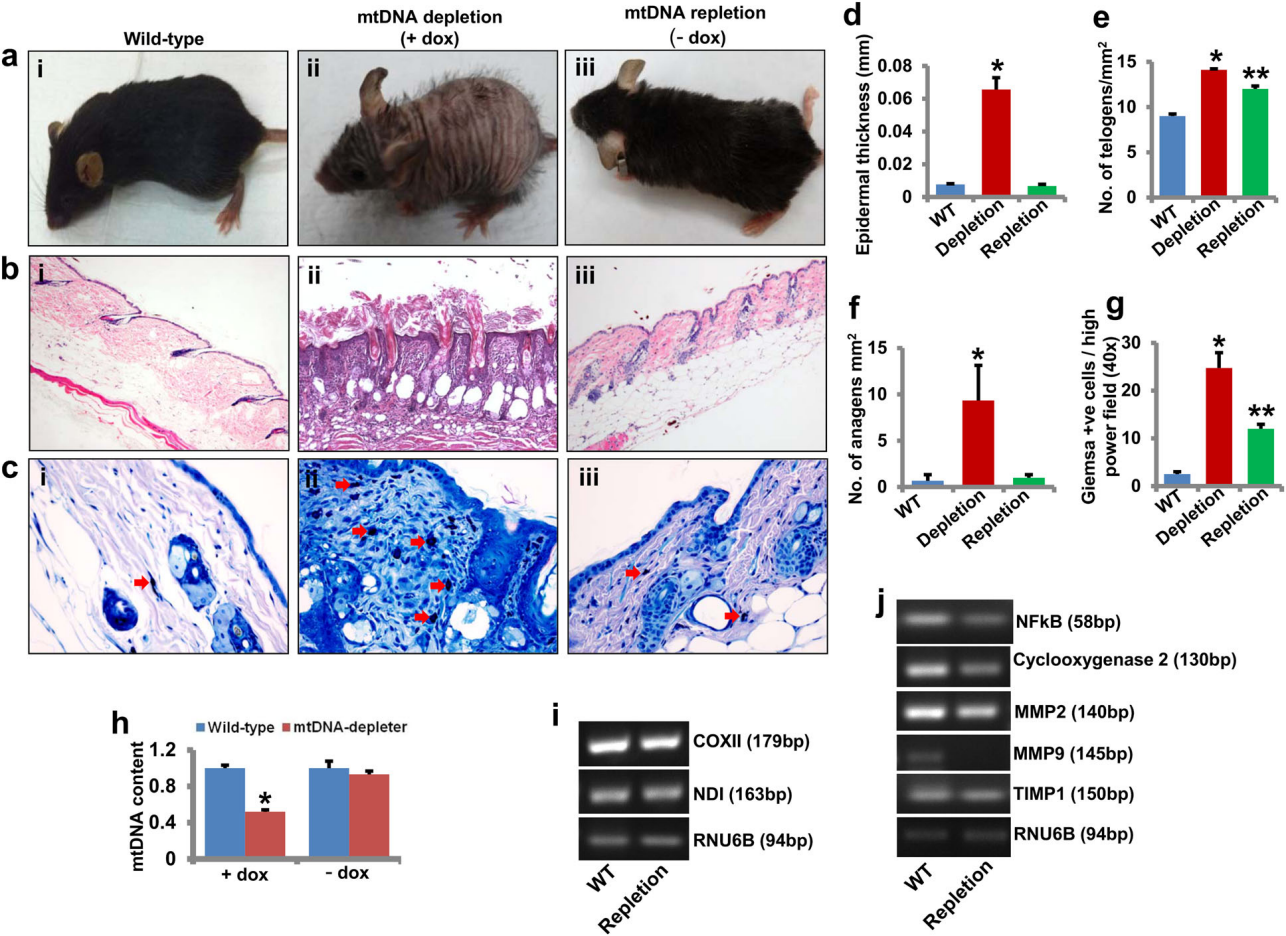
Reversal of wrinkled skin and hair loss to wild-type by restoration of mitochondrial function. **a** Representative images of a mtDNA-depleter mouse showing skin wrinkles and hair loss after 2 months of continuous dox induction (+dox; mtDNA depletion) (ii) and reversal of these phenotypic changes after 1 month of dox withdrawal (−dox; mtDNA repletion) (iii; *n* **=** 3). Wild-type control mice (*n* = 3) did not show any change in skin phenotype after dox treatment or after 1 month of dox withdrawal (i). **b** Hematoxylin- and eosin-stained sections of dorsal skin from wild-type control mice (i), mtDNA-depleter mice after 2 months of continuous dox induction (mtDNA depletion) (ii) and after 1 month of dox withdrawal (mtDNA repletion) (iii). **c** Giemsa staining of skin sections shows the presence of increased number of mast cells in the dermis and perifollicular as well as near-epidermal locations of mtDNA-depleter mice compared with skin sections of wild-type mice. Compared with mtDNA-depleter mice, the lower number of mast cells is present in the skin of mtDNA-depleter mice after 1 month of dox withdrawal (−dox; mtDNA repletion). Arrows indicate the presence of Giemsa-positive cells in the skin sections. **d**–**f** Quantification of epidermal thickness (**d**), hair follicles in telogen (**e**), and anagen (**f**) stages of hair cycles (mean ± s.e.m; *P<0.05, Student’s *t* test) in skin samples from wild-type control (*n* = 3) and mtDNA-depleter (*n* = 3) mice after 2 months of continuous dox induction (mtDNA depletion) and after 1 month of dox withdrawal (mtDNA repletion). **g** Quantitative analysis of Giemsa-positive mast cells in the skin sections of wild-type control, mtDNA-depleter mice after 2 months of continuous dox induction (mtDNA depletion), and after 1 month of dox withdrawal (mtDNA repletion) (mean ± s.e.m; *P<0.05, Student’s *t* test). **h** Quantification of mtDNA content (mean ± s.e.m; *P<0.05, Student’s *t* test) in skin samples from wild-type control (*n* = 3) and mtDNA-depleter (*n* = 3) mice after 2 months of continuous dox induction and reversal of mtDNA content in skin samples of mtDNA-depleter mice (*n* = 3) after 1 month of dox withdrawal. **i**, **j** Representative gel images showing RT-PCR analysis of genes in the skin RNA samples of wild-type control (*n* = 3) and mtDNA-depleter mice (*n* = 3) after 1 month of dox withdrawal (mtDNA repletion). Depletion = mtDNA depletion, repletion = mtDNA repletion

We conducted rescue experiment to substantiate that the mitochondrial dysfunction was the underlying cause for the alterations in the skin of mtDNA-depleter mice. Dox withdrawal restored mtDNA content to normal level in mtDNA-depleter mice. There was the induction of typical skin wrinkles and loss of hair in mtDNA-depleter mice (as shown in Fig. 9aii) after exposure to dox for 2 months. Then, after 1 month of dox withdrawal, the skin wrinkles and hair loss reverted, and the animals appeared relatively normal when compared to the age-matched wild-type animals (Fig. 9a). The histopathological analysis of the skin of phenotype-reversed (mtDNA-repleter) animals showed restoration of normal cutaneous structures (Fig. 9b). The epidermal hyperplasia (Fig. 9d), abnormal sebaceous glands, and defects in hair follicle development and hair shaft formation were absent in the mtDNA-repleter mice (Fig. 9b). The number of anagen hair follicles reverted to the wild-type levels (Fig. 9f), and the number of hair follicles in telogen also decreased in the mtDNA-repleter mice compared with mtDNA-depleter mice (Fig. 9e). We also observed a significant decrease in the inflammatory infiltrate present in the skin of phenotype-reversed animals (Fig. 9b, c, g). The macrophages, granulocytes, and B lymphocyte and T lymphocyte that were present in the skin of mtDNA-depleter mice (Fig. 7a) were predominantly absent in the skin of the mtDNA-repleter mice (data not shown). We observed a reversal of mtDNA content (Fig. 9h) and the expression of mtDNA-encoded genes (Figs. 2d and 9i). Expression of genes involved in the skin inflammation and wrinkling also reverted to the levels in wild-type animals (Figs. 7c, d and 9j). These observations suggest that mitochondrial dysfunction-induced phenotypical, histopathological, and molecular changes can be reversed by restoration of mitochondrial function.

## Discussion

Accumulating evidence suggests a strong link between mitochondrial dysfunction, mitochondrial diseases, aging, and aging-associated diseases^26, 28, 30, 38, 63^. Notably, increased somatic mtDNA mutations and decline in mitochondrial functions have been extensively reported during human aging^26, 28, 30^. Studies also suggest a decrease in mtDNA content and mitochondrial number with age^32, 33, 64^. The major finding of our study is that the ubiquitous depletion of mtDNA predominantly leads to wrinkled skin and hair loss accompanied by inflammatory phenotype. Wrinkled skin and hair loss are obvious features of skin aging and aging-associated phenotypic changes in humans. We discovered that these aging-associated phenotypic changes could be reversed by restoring mtDNA content to wild-type level. To our knowledge this observation is unprecedented.

Skin wrinkles are a hallmark of both intrinsic and extrinsic aging of the skin in humans^65^. Mitochondrial dysfunction is implicated in both intrinsic and extrinsic aging^54, 55, 64^. The presence of skin wrinkles, acanthosis, epidermal hyperplasia with hyperkeratosis, and marked inflammatory infiltrate in the skin of mtDNA-depleter mice (Figs. 6a and 7a, b) represent characteristics similar to the extrinsic aging of skin in humans^55^. Furthermore, the changes in expression of intrinsic aging-associated genetic markers support intrinsic mechanisms underlying this phenotypic change in mtDNA-depleter mice (Fig. 8). In support of our studies, a link between accumulation of mtDNA mutations and aging phenotype in the mouse has been identified previously^37, 38, 54^.

Loss of collagen fibers is reported to underlie skin wrinkles^58^. A tight balance between the proteolytical enzymes MMPs and their tissue-specific inhibitor TIMP1 is essential to maintain the collagen fiber content in the skin^59^. Expression of MMPs is altered in the aged skin^66, 67^. Consistent with these reports, the skin of mtDNA-depleter mice showed increased expression of MMPs and decreased expression of TIMP1, indicating loss of balance contributing to the development of skin wrinkles (Fig. 7d). Repletion of mtDNA content restored MMP expression (Fig. 9j) leading to a reversal of wrinkled skin and hair loss (Fig. 9a, b). These experiments show that mitochondria are regulators of skin aging and loss of hair. This observation is surprising and suggests that epigenetic mechanisms underlying mitochondria-to-nucleus cross-talk must play an important role in the restoration of normal skin and hair phenotype. Further experiments are required to determine whether phenotypic changes in other organs can also be reversed to wild-type level by restoration of mtDNA.

mtDNA stress triggers inflammatory response^54, 55, 68^. Inflammation also underlies aging and age-related diseases^67^. Increased level of markers of inflammation in our study indicates an activated immune response in the skin of mtDNA-depleter mice (Fig. 7a–c). Increased expression of NF-κB, a master regulator of the inflammatory response, upon mtDNA depletion (Fig. 7c) and its reduced expression after the restoration of mtDNA content (Fig. 9j) suggests that NF-κB signaling is a critical mechanism contributing to the skin and hair follicle pathologies in mtDNA-depleter mice. Indeed, NF-κB signaling was described as a well-known regulator of tissue homeostasis previously^69, 70^. Furthermore, a unique feature of proteins encoded by mtDNA is *N*-formyl-methionine at the N terminus^68^. *N*-formylated peptides when present in the extracellular space are known to act as mitochondrial damage-associated molecular patterns and activate neutrophils or activate keratinocyte-intrinsic responses resulting in the recruitment of immune cells^69, 70^. A recent study also supports our finding which shows that expression of mutant mitochondrial helicase (K320E-TWINKLE) in the mouse epidermis induces inflammation in the skin^68^. Despite these similarities at the level of skin inflammation between our results and this report^68^, we noticed major differences. Firstly, our study used whole-animal approach to ubiquitously deplete mtDNA to disrupt mitochondrial function instead of a targeted approach in the epidermis. Thus, our studies indicate an important role of mitochondria in the skin when compared to other tissues. Secondly, our studies demonstrate that reversal of inflammatory gene expression strongly suggest a role for epigenetics in the regulation of these genes. Lastly, we demonstrate clearance of infiltrated immune cells from dermis upon restoration of the mitochondrial function instead of otherwise massive immune cell presence in the skin of mutant TWINKLE mouse. Also, the short lifespan of K320E-Twinkle^epi^ mice prevented any aging study; however, we show the development of wrinkles and loss of hair, a persistent and profound feature of human aging. Similarly, epidermis-specific Tfam-knockout mouse shows defects in epidermal differentiation and hair follicle morphogenesis during embryonic development^71, 72^. However, due to a short life of Tfam-knockout mouse, these studies did not observe the effect of mitochondrial dysfunction leading to skin wrinkles and hair loss in adult mice^71, 72^.

In summary, development of mtDNA-depleter–repleter mouse revealed that the loss of mtDNA homeostasis is responsible for the development of skin wrinkles, epidermal hyperplasia, inflammatory phenotype, and loss of hair due to abnormal development of adnexal structures. This mouse allowed the ubiquitous suppression and restoration of mitochondrial function in the whole animal. The mtDNA-depleter mouse can help rapidly identify genes and pathways that can help in molecular understanding leading to amelioration of mtDNA diseases. Furthermore, this animal model holds promise to generate tissue-specific modulation of mitochondrial functions to determine, for various organs, the effects of mitochondria on in vivo aging, and pathogenesis of MDS and other mitochondrial diseases. Together, this mouse model should provide an unprecedented opportunity for the development of preventative and therapeutic drug development strategies to augment the mitochondrial functions for the treatment of aging-associated skin and hair pathology and other human diseases in which mitochondrial dysfunction plays a significant role.

## Materials and Methods

### Creation of mtDNA-depleter mice

D1135A-POLG1 site-directed mutation was created in the full-length human POLG1 complementary DNA (cDNA) using the site-directed mutagenesis kit (Agilent, Santa Clara, CA, USA). The primer sequences used for site-directed mutagenesis are as follows, with the mutated site in upper case: D1135A_F:5′-gcatcagcatccatgCGgaggttcgctacctgg-3′ and D1135A_R:5′-ccaggtagcgaacctcCGcatggatgctgatgc-3′. Mutations were confirmed by sequencing. D1135A-POLG1 cDNA was subcloned into the dox-inducible mammalian expression vector, pTRE-Tight-BI-AcGFP1 (Clontech, Palo Alto, CA, USA). To obtain germline transmission of human D1135A-POLG1 (POLG1-DN), microinjection of the pTRE-Tight-BI-AcGFP1-D1135A-POLG1 construct into fertilized oocytes from C57BL/6 mouse was carried out. Potential founders were identified by screening genomic DNA from tail biopsies for the presence of the human *Polg1* transgene using the PCR. The heterozygous human POLG1-positive (+/POLG1-DN^+^) founder male mice were mated with CAG-rtTA3 (rtTA) C57BL/6 female mice (Jackson Laboratories, stock no. 016532) to obtain +/POLG1-DN^+^ rtTA^+^ heterozygous transgenic mice. The +/POLG1-DN^+^ rtTA^+^ heterozygous mice were intercrossed to generate homozygous POLG1-DN^+^ rtTA^+^/POLG1-DN^+^ rtTA^+^ mice (mtDNA-depleter mice). This cross resulted in normal litter size (6–7 pups) and Mendelian distributions of genotypes, that is, 1:2:1 distribution of wild-type, heterozygous +/POLG1-DN^+^ or +/rtTA^+^ and homozygous POLG1-DN^+^ rtTA^+^/POLG1-DN^+^ rtTA^+^ showing that homozygosity for POLG1-DN allele does not result in embryonic or postnatal lethality. All animal experiments were conducted by following guidelines established by the Institutional Animal Care and Use Committee.

### Histological and immunohistochemical analyses

Skin from the dorsal side as well as other tissues was fixed in buffered formalin, embedded in paraffin, sectioned (5 µM), and stained with hematoxylin and eosin. Skin sections were stained with Giemsa stain to detect mast cells, while MPO, CD3, CD163, and Pax-5 antibodies were used for detection of other types of inflammatory cells by immunohistochemical analyses^73^.

### RT-PCR and mtDNA content analyses

To measure relative gene expression by RT-PCR, total cellular RNA from the skin samples was isolated using Trizol (Invitrogen, Carlsbad, CA, USA). Approximately, 1000–2000 ng RNA was normalized across samples, and cDNA was generated using the Iscript cDNA synthesis kit (Bio-Rad Laboratories, Hercules, CA, USA). cDNA was then subjected to RT-PCR using Green Taq PCR mixture (Promega, Madison, WI, USA) and gene-specific primers as given in Supplementary Table S1. PCR products were run on 1.5 to 2% agarose gel and photographed using gel documentation system. At least three biological replicates were used in each PCR. β2-Microglobulin or RNU6B was used as an internal control in each PCR. mtDNA content analyses in the skin and other tissues were carried out as reported earlier^74^.

### BN-PAGE and western blot analyses

Mitochondrial isolation was carried out as previously described^75^. To analyze mitochondrial OXPHOS supercomplexes, Blue-Native polyacrylamide gel electrophoresis (BN-PAGE) was performed with mitochondrial fractions prepared from the skin samples as described previously^76^. Protein expression of mitochondrial OXPHOS subunits in the skin samples was carried out following standard immunoblots. A premixed cocktail containing primary monoclonal antibodies (Mitosciences, Eugene, OR, USA) against subunits of OXPHOS complexes was used to detect OXPHOS supercomplexes in BN-PAGE analyses and protein expression of OXPHOS subunits in immunoblot analyses. Voltage-dependent anion channel (VDAC) or β-actin antibodies were used as loading controls.

### Analysis of enzymatic activities of OXPHOS complexes

Isolated mitochondria were used for the measurement of enzymatic activities of OXPHOS complexes as previously described^77^.

### Transmission electron microscopy

Transmission electron microscopic analyses of skin samples were carried as described previously^78^. Images were taken using the FEI-Tecnai electron microscope.

### Statistical analyses

Statistical analyses were performed using unpaired Student’s *t* test. Data are expressed as mean ± s.e.m. *P* values <0.05 were considered significant. All cellular experiments were repeated at least three times.

## Electronic supplementary material

Supplemental Material
