# Supplementary material for: Reversing wrinkled skin and hair loss in mice by restoring mitochondrial function
Source: Cell Death Dis. 2018 Jul 20;9(7):735. doi: 10.1038/s41419-018-0765-9 (PMC6053453; doi:10.1038/s41419-018-0765-9)
Supplement: Supplementary file 1 — Supplemental Material [file 41419_2018_765_MOESM1_ESM.doc]

**Supplementary Information**

**Reversing Wrinkled Skin and Hair Loss in Mice by Restoring Mitochondrial Function**

Bhupendra Singh, Trenton R. Schoeb, Prachi Bajpai, Andrzej Slominski, Keshav K. Singh


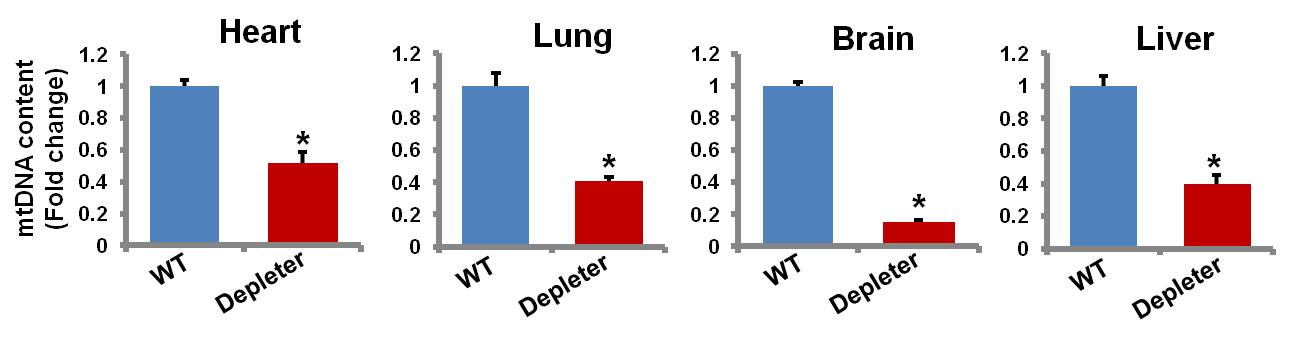


**Supplementary Figure S1. Doxycycline-mediated induction depletes mtDNA content in mtDNA-depleter mice**

Quantification of mtDNA content (mean ± s.e.m; asterisk, *P* <0.05, Student's *t*-test) in heart, lung, brain, and liver tissue samples of wild-type control (WT; *n* = 3) and mtDNA-depleter (Depleter; *n* = 3) mice after two months of continuous dox-induction.

**Supplementary Table S1. List of PCR primers used in the study**

| **Primers used for genotyping** | | |
| --- | --- | --- |
| Target | Forward primer | Reverse primer |
| POLG1 | CAA GGT CCA GAG AGA AAC TG | CTC TGT ACC ACC CAA TTC AC |
| CAG-rtTA | CTG CTG TCC ATT CCT TAT TC | CGA AAC TCT GGT TGA CAT G |
| GFP | GGG CAA TAA GAT GGA GTA CA | TGG ACA GGT AGT GGT TAT CG |
| **Primers used for RT-PCR** | | |
| POLG1 | CCA GGG AGA GTT TAT AAC CA | CAA ATT CCT CAA ACA GCC AC |
| COXII | GGC ACC TTC ACC AAA ATC AC | CGG TTG TTG ATT AGG CGT TT |
| NDI | CCT ATC ACC CTT GCC ATC AT | TTG CTG CTT CAG TTG ATC GT |
| NF-κB | TGG CCG TGG AGT ACG ACA A | GCA TCA CCC TCC AGA AGC A |
| MMP2 | ACC TGA ACA CTT TCT ATG GCT G | CTT CCG CAT GGT CTC GAT G |
| MMP9 | CTG GAC AGC CAG ACA CTA AAG | CTC GCG GCA AGT CTT CAG AG |
| TIMP1 | CTT GGT TCC CTG GCG TAC TC | ACC TGA TCC GTC CAC AAA CAG |
| COL1A1 | CTG GCG GTT CAG GTC CAA T | TTC CAG GCA ATC CAC GAG C |
| Cyclooxygenase 2 | AAC CGC ATT GCC TCT GAA T | CAT GTT CCA GGA GGA TGG AG |
| CCL5 | AGA TCT CTG CAG CTG CCC TCA | GGA GCA CTT GCT GCT GGT GTA G |
| IL28a | AGG TCT GGG AGA ACA TGA CTG | CTG TGG CCT GAA GCT GTG TA |
| IFNB1 | GTC ATG GGT TTC TCA TGA AGA ACA G | CAG ACC CCT TCC AGT GAT TCA TC |
| VEGF | GAG GAT GTC CTC ACT CGG ATG | GTC GTG TTT CTG GAA GTG AGC AA |
| IGF1R | CGA GCT TCC TGT GAA AGT GAT GT | CAC GTT ATG ATG ATT CGG TTC TTC |
| Klotho | GGA CAT TTC CCT GTG ACT TTG C | AGA GAG AGT AGT GTC CAC TTG AAC GT |
| MRPS5 | AAC CAC TGT CTG ACC AGC TTG | AGT CTC TGC TAA TGC GCC TTT |
| RNU6B | CTC GCT TCG GCA GCA CA | AAC GCT TCA CGA ATT TGC GT |
| B2M | ATG GGA AGC CGA ACA TAC TG | CAG TCT CAG TGG GGG TGA AT |
